# Obesity decreases both whole muscle and fascicle strength in young females but only exacerbates the aging‐related whole muscle level asthenia

**DOI:** 10.14814/phy2.12030

**Published:** 2014-06-24

**Authors:** David J. Tomlinson, Robert M. Erskine, Keith Winwood, Christopher Ian Morse, Gladys L. Onambélé

**Affiliations:** 1 Department of Exercise and Sport Science, Institute for Performance Research, Manchester Metropolitan University, Crewe Green Road, Crewe, CW1 5DU, U.K; 2 Research Institute for Sport and Exercise Sciences, Liverpool John Moores University, Liverpool, L3 3AF, U.K

**Keywords:** Aging, obesity, specific force

## Abstract

Obesity has previously been associated with greater muscle strength. Aging, on the other hand, reduces muscle specific force (the force per unit physiological cross‐sectional area [PCSA] of muscle). However, neither the effect of obesity on skeletal muscle specific force nor the combined effects of aging and obesity on this parameter are known. This study aimed to describe the interplay between body mass index (BMI)/adiposity, aging, and skeletal muscle specific force. Ninety‐four untrained healthy women categorized by age into young (Y; mean ± SD: 25.5 ± 9.0 years) versus old (O; 64.8 ± 7.2 years) were assessed for body composition, gastrocnemius medialis (GM) muscle volume (*V*), net maximum voluntary contraction (nMVC), and specific force (SF). The young obese, while demonstrating 71% and 29% (*P* < 0.001) higher *V* and nMVC compared to normal BMI individuals, were in fact 26% (*P* = 0.007) weaker than these, where *V* was used to scale nMVC (i.e., nMVC/*V*). The weakness associated with obesity was further exemplified in the 34% (*P* < 0.001) lower SF relative to normal BMI individuals. Similarly, ≥40% body fat was associated with 60% and 27% (*P* < 0.001) higher *V* and nMVC, but 11% and 25% (*P* < 0.01) lower nMVC/*V* and SF than <40% body fat. The aging‐related rates of decline in *V* (−2 cm^3^/year *P* < 0.05) and nMVC (−1.2 cm^3^/year *P* < 0.05) were highest in obesity defined by BMI. This effect was also seen when segregating by >40% adiposity. Interestingly, however, obesity appeared advantageous to the aging‐related changes in nMVC/*V* (*P* < 0.001) and SF (*P* < 0.001). Unlike previous reports of greater strength in the obese compared with leaner age‐matched counterparts, we in fact demonstrate that the young sedentary obese, are substantially weaker, where the volume of skeletal muscle is used to scale the maximal torque output, or forces are quantified at the fascicular level. The seemingly positive impact of obesity on rate of aging, however, is complex and warrants further investigations.

## Abstract (precis)

Our study demonstrates for the first time that at both whole muscle and fascicular levels, high body mass index (BMI) or adiposity categories of obesity are associated with significantly lower skeletal muscle contractile capacity in young adults. Interestingly, the aging effect on obese individuals classified by both BMI and adiposity was foremost observed through the loss of muscle tissue content as well as total muscle strength.

## Introduction

It is generally accepted that obese individuals, regardless of age, have lower maximal strength when the latter is expressed relative to body mass (Blimkie et al. 1990; Hulens et al. 2001; Rolland et al. 2004; Lafortuna et al. 2005; Maffiuletti et al. 2007, 2008; Abdelmoula et al. 2012). However, it is unclear whether this weakness exists at the fascicle level, or is simply a reflection of pseudo‐hypertrophy whereby the relative high amount of muscle mass in an obese person is superseded by the higher elevation in adiposity. Previous studies have shown that the maximum strength capability relative to muscle size/mass of an obese individual is not significantly different to that of normal weight individuals (Blimkie et al. 1990; Lafortuna et al. 2005; Maffiuletti et al. 2007, 2008). This, however, could be a potential type II error due to the use of total fat‐free mass (Lafortuna et al. 2005; Maffiuletti et al. 2007, 2008) and anatomical cross‐sectional area (ACSA; Blimkie et al. 1990) as indices of muscle size. This can be explained by the aforementioned measures not accounting for the architectural characteristic of the skeletal muscle responsible for the joint action, thus potentially misconstruing the true amount of muscle that contributes to torque production (Alexander and Vernon 1975). However, contradictions also exist in the literature with previous authors (Hulens et al. 2001) reporting that obese adult individuals have 6–7% lower torque relative to total fat‐free mass and hypothesizing that this effect may be due to an assumed reduced agonist muscle activation. Indeed lower agonist muscle activation has been recorded in both obese adolescent (Blimkie et al. 1990) and obese adult populations (Tomlinson et al. 2014). Contrary to the above, other authors (Abdelmoula et al. 2012) reported obese adolescents to have 22% higher knee extension torque normalized to thigh lean muscle mass (data obtained from dual energy x‐ray absorptiometry [DEXA]). In fact, the only study to have utilized a more accurate and valid method of measuring the muscle mass involved in isometric torque production was that of Hilton et al. (2008) who assessed the muscle volume of the triceps surae in obese individuals. These authors (Hilton et al. 2008) demonstrated that obese individuals produced lower torque relative to muscle volume. However, the participants recruited had diabetes mellitus and peripheral neuropathy, both of which have previously been shown to independently cause motor weakness (Andersen et al. 1996).

To our knowledge, to date, no obesity/strength interaction study has accounted for the pennate architecture of the antigravity musculature of the lower limbs in the estimation of muscle size. An accurate quantification of muscle size is its physiological cross‐sectional area (PCSA; volume ÷ fascicle length), as this represents the number of sarcomeres in parallel, and exhibits a linear association with a muscle's maximum force capability (Fukunaga et al. 2001). Skeletal muscle specific force (force per unit PCSA) depicts an accurate representation of a muscle's maximum strength capacity, as it corrects for both the physiological and biomechanical determinants of maximal muscle strength (Maganaris et al. 2001; Reeves et al. 2004b; Erskine et al. 2009).

Aging has been shown to reduce skeletal muscle specific force (Morse et al. 2005). This reduction has been hypothesized as being due to a decline in the muscle activation capacity of elderly individuals, partly owing to lower habitual physical activity levels (Morse et al. 2004). In addition, preferential muscle fiber atrophy (Lexell and Taylor 1991) and a decrease in number of type II fibers (Lexell 1995) accompanied with an increase in intramuscular fatty infiltration (Rice et al. 1989), are likely to play a role in the aging‐related lowering of muscle specific force. Yet, it appears that no investigation has systematically examined whether, after accounting for neural and architectural factors underlying muscle force production, the effects of aging would be exacerbated in the presence of obesity, thereby leading to a greater drop in maximal force generation capability of the old obese compared to their normal weight, age‐matched counterparts. The increased prevalence of obesity (James 2008), which accompanies the rising level in life expectancy, renders the determination of therapeutic interventions to reverse the deleterious effects of the two “conditions” extremely timely.

Therefore, the aim of the present study was to investigate the degree of impact of obesity on skeletal muscle intrinsic force at both whole muscle and fascicular levels, in a young sedentary population. The second aim was to determine whether the effects of aging and adiposity are in fact additive on skeletal muscle specific force at both whole body and fascicle levels. We hypothesized that (1) skeletal muscle specific force in both obese Y and O would be lower when compared to lean, normal weight and overweight individuals, where muscle strength is quantified at both gross and fascicular levels; (2) the deleterious impact of high adiposity on skeletal muscle specific force (at both gross and fascicular levels), would be worse in the older individuals and their younger counterparts; and (3) the rate of aging (decrease in muscle contractile capacity) would be faster in the presence of obesity.

## Methods

### Ethical approval

Participants gave written informed consent prior to undertaking any assessment. All the procedures in this study had approval from the Manchester Metropolitan University Ethics committee and conformed to the standards set by the latest revision of the Declaration of Helsinki.

### Participants

The current study was on a single gender basis in order to minimize and the potential confounding effect of male versus female differential rate of aging (Lindle et al. 1997), and/or sensitivity to adiposity (Lafortuna et al. 2005, 2013). Thus, 94 untrained females volunteered to take part in this study (Table 1) and were categorized by age into either young (Y; 18–49 years) or old (O; 50–80 years). As 91/94 participants were white Caucasians, no subgrouping by ethnicity was carried out. Participants were then subcategorized into four body mass index classifications (BMI – body mass [kg]/stature^2^ [m]) into underweight (BMI <20), normal (BMI 20–24.9), overweight (BMI 25–29.9), and obese (BMI >30). Participants were also categorized as ordinary adipose (<40%) versus high adipose (≥40%) by body fat percentage following recommendations from previous studies (Baumgartner et al. 2004; Rolland et al. 2009). The exclusion criteria were any health issues highlighted in the self‐report questionnaire such as lower limb muscles/joints injuries/pathology, affecting mobility or ability to exert maximal torque. Physical activity status was screened by questionnaire and participants were excluded if they self‐reported as habitually undertaking structured exercise for more than 3 h per week.

**Table 1.** Descriptive variables for body mass index (BMI) classifications in both the young (upper section) and old (lower section) age classifications.

	Underweight	Normal	Overweight	Obese	BMI effect	Aging effect
*n*						
Young	13	12	8	16		
Old	4	14	16	11		
BMI (kg/m^2^)						
Young	18.8 ± 0.9	21.6 ± 1.1	27.8 ± 0.7	35.2 ± 4.5	*P* < 0.001	
Old	19.1 ± 0.8	22.2 ± 1.0	27.4 ± 1.2	34.1 ± 5.7	*P* < 0.001	*P* = n.s.
%Body fat						
Young	26.5 ± 3.9	30.1 ± 3.5	37.7 ± 5.8	45.5 ± 4.1	*P* < 0.001	
Old	26.5 ± 2.1	35.6 ± 3.4	43.3 ± 2.8	46.1 ± 5.0	*P* < 0.001	*P* = 0.002
Aging slope intrinsic strength (Nm/*V*.year^−1^)	0.028 (*P* = n.s.)	−0.347 (*P* = n.s.)	−0.261 (*P* < 0.01)	0.085 (*P* = n.s.)		
Aging slope‐specific force (N/cm^2^.year^−1^)	0.028 (*P* = n.s.)	−0.002 (*P* = n.s.)	0.045 (*P* = n.s.)	0.194 (*P* = n.s.)		

Data are presented as mean ± SD. Rate of aging per BMI classification is also shown (data are presented as regression slope and *P* value for the degree of association).

### Protocol order

Participants attended the laboratory for testing on two occasions. During the first visit, anthropometric measurements (DEXA, Stature, Mass) were collected, and familiarization with the MVC protocols took place. During the second visit participants gastrocnemius medialis muscle volume and architecture data were collected alongside the main MVC protocol.

### Body composition measure

**Figure 1.**
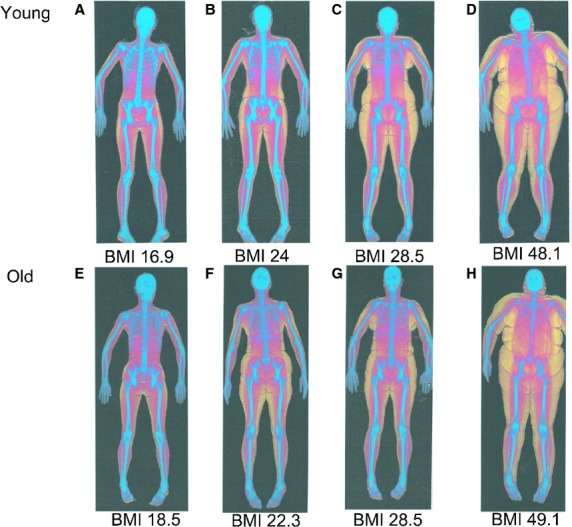
Representative dual energy x‐ray absorptiometry (DEXA) scans of a (A) young underweight female, (B) young normal weight female, (C) young overweight female, (D) young obese female, (E) old underweight female, (F) old normal weight female, (G) old overweight female and (H) old obese female. Color key: blue for bone; red for lean tissue; yellow for adipose tissue.

Body composition (body fat percentage, lean muscle, and bone) was ascertained using a DEXA scanner (Hologic Discovery; Vertec Scientific Ltd, Reading, U.K.) following a period of overnight fasting for 12 h. Participants lay in the supine position, avoiding any contact between the trunk and the appendicular mass during a 7‐min scanning procedure (whole body procedure, effective dose 8 *μ*Sv). Scan results were both graphical (Fig. 1) and numerical, giving a number of descriptions of which %body fat was key for the aims in this study.

### Muscle strength measurement

Maximum voluntary contraction (MVC) torque during both ankle plantar flexion (PF) and dorsi flexion (DF) was measured in the dominant limb using an isokinetic dynamometer (Cybex Norm; Cybex International, New York, NY). Participants were seated (hip at 85° angle, dominant leg extended with the foot secured to the footplate of the dynamometer), and strapped using inextensible straps (at the hip, distal thigh, and chest) to reduce extraneous movements. Prior to MVCs, participants were refamiliarized with the protocol undertaken during the test. Following this familiarization protocol, participants conducted a series of five submaximal isometric contractions with their ankle positioned at 0° (anatomically neutral), starting at self‐perceived 50% maximal exertion, increasing in intensity to ensure the participant was warmed up prior to maximal exertion.

**Figure 2.**
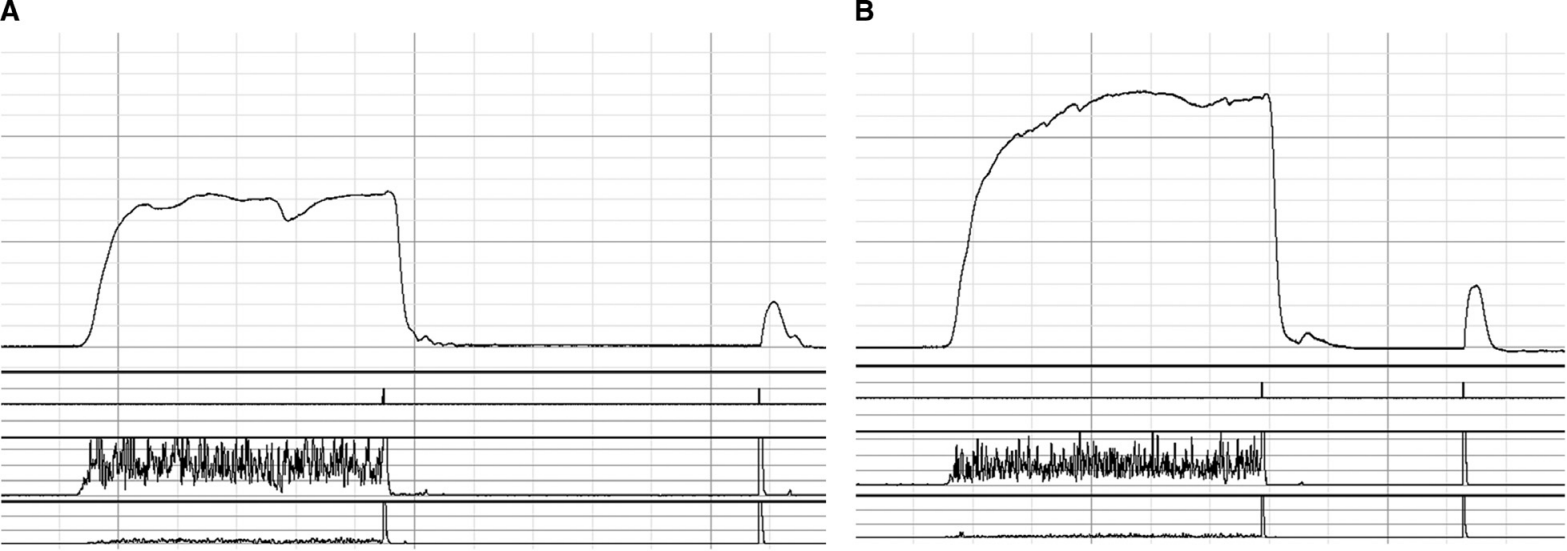
Representative torque outputs taken during the interpolated twitch technique of a (A) young ordinary‐adipose female and (B) young high‐adipose female.

During the main MVC protocol, participants were asked to conduct two (up to a maximum of four, see below) rapid isometric PF and DF MVCs with their ankle positioned at 0°, each lasting 3–4 sec. The highest (of two) recorded PF and DF MVC by the participant was utilized as their true MVC. However, MVCs were repeated if there was >10% difference between MVCs to ensure true MVC was obtained. The PF MVC was then corrected for agonist muscle activation using the interpolated twitch technique (Morse et al. 2004; Pearson and Onambele 2006; Fig. 2) and antagonist cocontraction of the tibialis anterior (TA) using surface electromyography (EMG). Antagonist muscle cocontraction was calculated through utilizing the EMG signal (computed as root mean square) of the TA recorded 500 msec on either side of instantaneous peak torque during a maximal PF and divided by the EMG recorded during DF. The raw EMG signal measured during contractions was recorded at 2000 Hz, with band pass filter set at 10–500 Hz, and notch at 50 Hz. This calculation method assumes that the DF EMG/Torque relationship is linear (Maganaris et al. 1998). MVCs corrected for both agonist muscle activation and antagonist co‐contraction (i.e., neural factors) in the manuscript are classified as net MVC (nMVC).

### Muscle volume and intrinsic strength

Participants lay in the prone position with their ankle positioned at 0°. B‐mode ultrasonography was then used to ascertain the origin and insertion of the GM, where discrete muscle sites (25%, 50%, and 75% of length) were marked from the medial to lateral border of the GM. Thin strips (2 mm) of micropore tape (3M, Bracknell, Berkshire, U.K.) were placed axially 3–4 cm apart transversally along the nominated muscle lengths. The micropore tape was utilized as echo‐absorptive marker in the formation of ACSAs from the corresponding muscle lengths when using the photo‐editing software (Adobe Photoshop Elements; Version 10, Adobe Systems Incorporated, San Jose, CA). During recording of the ACSAs, the ultrasound probe (7.5 MHz linear array probe, 38‐mm wide) was held perpendicular to the GM on its medial border and moved along a designated marked pathway to its lateral border. The probe was moved steadily across the leg with constant light pressure to avoid compression of the muscle during scanning. This procedure was repeated twice at each discrete muscle site for reliability purposes. The construction of the ACSAs were undertaken using Adobe Photoshop (Version 10), where still transverse images at each individual muscle length were reconstructed using the micropore tape as anatomical markers in combination with anatomical landmarks along the GM muscle length. Following construction of the three individual ACSAs, the areas of the complete transverse ACSAs were undertaken using the analysis software ImageJ (1.45s; National Institutes of Health, Bethesda, MD). Reliability in the measure of the three ACSAs was assessed in 10 participants (Y = 5; O = 5; BMI range = 17.6–36.7) on two separate days (separated by at least 48 h) by the same investigator. The intraclass coefficients were as follows: GM ACSA 25% length – 0.998, GM ACSA 50% length – 0.999, GM ACSA 75% length – 0.998. These measures are not only reliable but also externally valid as they demonstrate strong agreement with MRI‐obtained values (Reeves et al. 2004a). Muscle volume was then calculated using the truncated cone method:
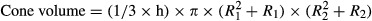


Where *R*_1_ = radius of the base; *R*_2_ = radius of the top; *h* = distance between segments; *R* = √(ACSA/*π*), where *π *= 3.142.

Skeletal muscle intrinsic strength was quantified as nMVC relative to muscle volume.

### Calculation of GM‐specific force

This involved several steps including the assessment of tendon force (itself requiring measurement of tendon moment arm) and muscle physiological cross‐sectional area (itself requiring the assessment of muscle architecture).

#### Tendon moment arm

Tendon excursion using B‐mode ultrasonography was used to calculate the Achilles tendon moment arm length (Ito et al. 2000; Maganaris et al. 2000). Participants were seated in the isokinetic chair (Cybex Norm; Cybex International) following the experimental set up undertaken during MVC's. Prior to commencement of the protocol, the insertion of the GM muscle to the Achilles tendon was anatomically marked on the limb using micropore tape as an echo‐absorptive reflective marker during recording. The ultrasound probe was then positioned across the muscle–tendon junction (MTJ) of the GM as denoted by the micropore tape. During the recording the ankle of the participant was passively rotated between 10° and −5° PF at a constant velocity of 1°/sec. The passive movement was recorded for at least three PF and DF rotations and was synchronized with torque outputs using a square wave signal generator to distinguish joint position on the capturing software.

The displacement of the MTJ of the GM between 10° PF and −5° PF was calculated using the micropore tape as a distance marker using analysis software (ImageJ 1.45s; National Institutes of Health). The Achilles moment arm length at 0° was then calculated using the displacement of the MTJ divided by the displacement in the ankle angle during a complete rotation.
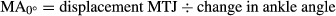


#### Tendon force

Achilles tendon force (*F*) at 0° was calculated using the PF MVC corrected for both agonist muscle activation and antagonist cocontraction, then dividing this value by the Achilles tendon moment arm length. The contribution of the GM muscle to PF MVC was calculated, assuming this muscle to contribute 25% of the total ankle plantar flexion MVC (Fukunaga et al. 1992).
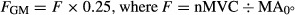


#### Muscle architecture

Muscle architecture of the gastrocnemius medialis (GM) was measured using B‐mode ultrasonography at both rest and during a graded isometric MVC over 6 sec. Participants were seated in an isokinetic dynamometer (Cybex Norm; Cybex International) as detailed above. The probe of a B‐mode ultrasound scanner (AU5 Harmonic; Esaote Biomedica, Genoa, Italy) was positioned on the surface of the skin, at 50% of the GM muscle length, along the mid‐sagittal line. Participants were then asked to perform a ramped MVC over 6 sec, where the change in both fascicle pennation angle (FPA) and fascicle length (Lf) were recorded. Images of both resting and maximal architecture were synchronized with torque outputs using a square wave signal generator, extrapolated from the capturing software (Adobe Premier pro Version 6; Adobe Systems Software, San Jose, CA) and later analyzed using ImageJ (1.45s; National Institutes of Health). Analysis of three fascicles defined from the deep to the superficial aponeurosis of the GM was then recorded and the mean value of both the FPA and Lf of the three fascicles were then recorded. If needed (in cases where the fascicles extended beyond the width of the probe), linear extrapolation of fascicles was carried out. The reliability of the measure of both FPA and Lf at rest and MVC was obtained from 10 participants (Y = 5; O = 5; BMI range = 17.6–36.7). The intraclass correlation coefficients for both the architectural measurements were high (muscle fascicle pennation angle rest – 0.997, muscle fascicle pennation angle max – 0.997, muscle fascicle length rest – 0.996, muscle fascicle length max – 0.993).

Following the calculation of GM muscle volume and architecture, PCSA was then calculated (PCSA = Muscle volume ÷ Lf).

#### Fascicle force

Following the computation of the GM muscle force (*F*_GM_), fascicle force was calculated as
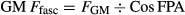


Gastrocnemius medialis muscle specific force was calculated by dividing the GM fascicle force by GM PCSA (Alexander and Vernon 1975; Fukunaga et al. 1996; Reeves et al. 2004b).
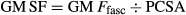


### Statistical analyses

Statistical analyses were carried out using SPSS (Version 19, SPSS Inc., Chicago IL). Stem‐and‐Leaf plots were used to identify any outliers, and these were removed prior to further analyses. To determine parametricity, Shapiro–Wilk (normal distribution) and Levene's tests (homogeneity of variance) were used. If parametric assumptions were met, a one‐way analysis of variance (ANOVA; BMI classifications) with post hoc Bonferroni correction for pairwise comparisons, or independent sample *t*‐tests (%body fat classifications) were used on muscle volume, nMVC, GM intrinsic strength, and GM specific force (GM SF). Where parametric assumptions were breached, Kruskal–Wallis *H* (BMI classifications) or Mann–Whitney *U* (%body fat classifications) were used. Linear regressions and Pearson's moment correlations, described the relationships and the degree of association, between age and parameters of interest (including muscle volume, nMVC). Comparisons of the regression coefficients and slopes were conducted using *z*‐transformations and the Student's *t*‐statistic. Data are reported as mean ± SD and statistical significance was accepted when *P* ≤ 0.05.

## Results

### Body composition

Tables 1 and 2 display descriptive study population characteristics of BMI and body fat% for Y and O females categorized by both BMI and %body fat.

**Table 2.** Descriptive variables for obesity classification by body fat percentage in both the young (upper section) and old (lower section) age classifications.

	Ordinary adipose (*n* = 32)	High adipose (*n* = 17)	Obesity effect	Aging effect
*n*				
Young	32	17		
Old	19	26		
BMI				
Young	21.9 ± 3.9	34.5 ± 5.0	*P* < 0.001	
Old	22.6 ± 3.5	29.6 ± 5.3	*P* < 0.001	*P* = 0.463
%Body fat				
Young	29.9 ± 5.1	45.9 ± 3.5	*P* < 0.001	
Old	33.6 ± 4.7	44.8 ± 3.7	*P* < 0.001	*P* = 0.006
Aging slope intrinsic strength (Nm/*V*.year^−1^)	−0.278 (*P* < 0.01)	−0.018 (*P* = n.s.)		
Aging slope‐specific force (N/cm^2^.year^−1^)	−0.033 (*P* = n.s.)	0.173 (*P* = n.s.)		

Data are presented as mean ± SD. Rate of aging per adiposity classification is also shown (data are presented as regression slope and *P* value for the degree of association).

### Skeletal muscle characteristics

#### BMI and muscle contractile characteristics

**Figure 3.**
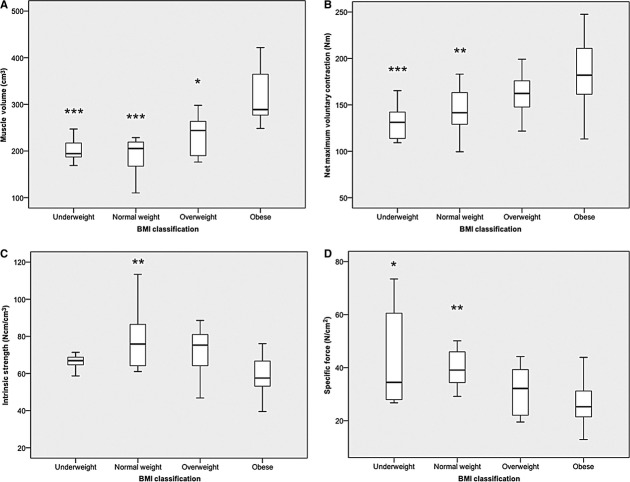
Displays the impact of body mass index (BMI) classification on (A) muscle volume (−1 overweight and 2 obese outliers), (B) net maximum voluntary contraction (0 outliers), (C) intrinsic strength (−3 underweight and 2 normal weight outliers) and (D) specific force (−1 normal weight outlier) in young females (*P < 0.05; *P < 0.01; ***P < 0.001).

Figure 3 demonstrates the effect BMI classification has upon muscle volume, nMVC torque, intrinsic strength, and specific force in young participants.

Muscle volume revealed a main effect of BMI classification (*P* < 0.001). Pairwise comparisons revealed the obese females to have 75% (*P* < 0.001), 71% (*P* < 0.001), and 36% (*P* < 0.010) greater muscle volume than their underweight, normal weight and overweight counterparts, respectively.

Net MVC torque revealed a main effect of BMI classification (*P* < 0.001). Pairwise comparisons revealed the obese females to have 39% (*P* < 0.001) and 29% (*P* = 0.005) greater nMVC than their underweight and normal weight counterparts, respectively.

The opposite ranking order was found for indices of strength normalized for muscle content. Indeed intrinsic strength revealed a main effect of BMI classification (*P* = 0.005). Pairwise comparisons revealed the obese females to have 26% (*P* = 0.007) lower intrinsic strength than their normal weight counterparts. However, there was no difference in intrinsic strength between the young obese and either underweight, or overweight individuals (Fig. 3C).

Skeletal muscle specific force (GM SF) revealed a main effect of BMI classification (*P* = 0.002). Pairwise comparisons revealed the obese females to have 40% (*P* = 0.006), 34% (*P* = 0.010) lower specific force than their underweight and normal weight counterparts, respectively. There was no difference in GM SF between overweight and obese young individuals.

#### Adiposity and muscle contractile characteristics

**Figure 4.**
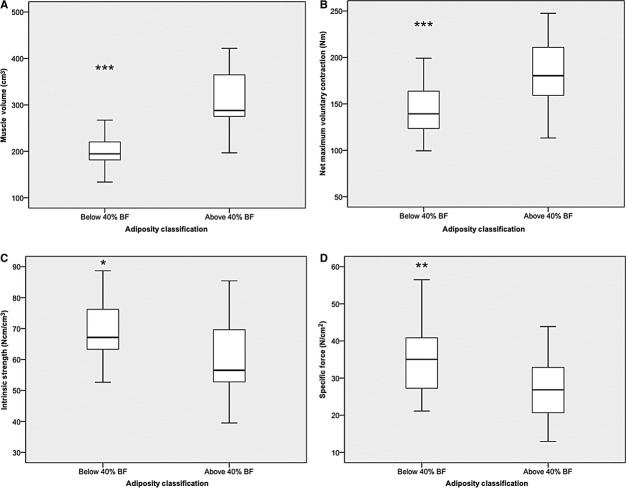
Displays the impact of adiposity on (A) muscle volume (−1 overweight and 1 obese outliers), (B) net maximum voluntary contraction (−1 overweight outlier), (C) intrinsic strength (−3 underweight and 2 normal weight outliers) and (D) specific force (−1 underweight, 1 normal weight, and 1 overweight outliers) in young females (*P < 0.05; *P < 0.01; ***P < 0.001).

Figure 4 demonstrates the effect that obesity classification by adiposity (i.e., %body fat) has upon muscle volume, nMVC, intrinsic strength, and specific force in young participants.

Highly adipose females were found to have 60% (*P* < 0.001) greater muscle volume, and 27% (*P* < 0.001) greater nMVC than “ordinary” adipose females.

Interestingly, however, as seen in BMI classifications, this seeming advantage of high adiposity was reversed when it was shown that the high‐adipose females had in fact 11% (*P* = 0.025) lower intrinsic strength, and 25% (*P* = 0.005) lower specific force than their age‐matched ordinary adipose‐matched females than the ordinary classified females.

#### Degree of association between age and muscle size and/or strength by obesity status

**Figure 5.**
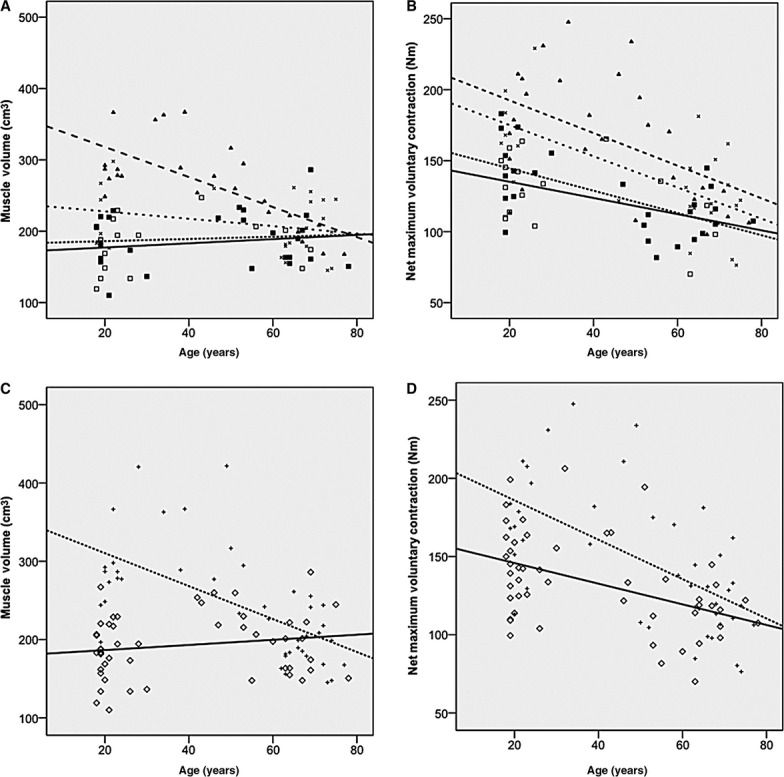
Displays linear regressions (and slope comparisons) on the impact of age on both BMI and adiposity classification on muscle volume (A, C) and net maximum voluntary contraction (B, D) in sedentary females. (A) Underweight (□ **——**) versus Obese (▲ — —) *P *<**0.05. Normal weight (■ ‐ ‐ ‐ ‐ ‐) versus Obese; *P *<**0.05. Overweight (× – – –) versus Obese *P *<**0.05**.** (B) Underweight (□) versus Obese (▲) *P *>**0.05; Normal weight (■) versus Obese; *P *>**0.05; Overweight (×) versus Obese *P *>**0.05. (C) Ordinary adipose ◊ ——; high adipose + ‐ ‐ ‐ ‐ ‐ *P *<**0.05; (D) Ordinary adipose versus high adipose; *P *>**0.05. Briefly, in terms of muscle volume, the obese individuals were shown to significantly decrease muscle mass per year at a fast rate (−2.1 cm^3^/year, *P *<**0.001) than overweight (−0.5 cm^3^/year *P *= n.s), normal weight (0.2 cm^3^/year, *P *= n.s), underweight females (0.3 cm^3^/year, *P *= n.s) or ordinary adipose females (−2.1 cm^3^/year *P *<**0.001 vs. −0.3 cm^3^/year *P *= n.s). In terms of nMVC, there was a (nonsignificant) trend for the obese individuals to lose maximum strength capability at a slightly greater rate (−1.2 Nm/year, *P *=**0.019) than their overweight (−1.1 Nm/year, *P *=**0.002), normal weight (0.8 Nm/year, *P *=**0.008), underweight females (0.6 Nm/year, *P *= n.s) or ordinary adipose females counterparts (−1.3 Nm/year, *P *<**0.001 vs. 0.7 Nm/year, *P *=**0.001).

There was a stepwise increment in the steepness of the aging versus muscle content loss relationship, with increasing BMI. Thus, aging‐related muscle loss from the second to the seventh decade were −2, −0.5, 0.2, and 0.3 cm^3^/year in the obese, overweight, normal weight, and underweight BMI categories, respectively. These differences in slopes were significant between obese and normal weight (Student's *t* statistic 3.88; *P* < 0.05), obese and underweight (Student's *t* statistic 3.64; *P* < 0.05), and obese and overweight (Student's *t* statistic 2.59; *P* < 0.05) females (Fig. 5A).

Similarly, there was a stepwise increment in the steepness of the aging versus muscle content loss, with high adiposity. Thus, aging‐related changes from the second to the seventh decade were −2.1 versus 0.3 cm^3^/year in high versus low %body fat categories. These differences in slopes were statistically significant (Student's *t* statistic 4.82; *P* < 0.05; Fig. 5C). Based on these slopes, it was evident that for muscle volume aging was associated with faster than normal deleterious changes, regardless of whether classification was by BMI or by fat.

There was a stepwise increment in the steepness of the aging versus nMVC loss relationship, with increasing BMI. Thus, aging‐related changes from the second to the seventh decade were −1.2, −1.1, 0.8, and 0.6 Nm/year in the obese, overweight, normal weight, and underweight BMI categories, respectively. These differences in slopes, were significantly different between obese and normal weight (Student's *t* statistic 3.88; *P* < 0.05), obese and underweight (Student's *t* statistic 3.64; *P* < 0.05), and obese and overweight (Student's *t* statistic 2.59; *P* < 0.05) females (Fig. 5A).

Similarly, there was a stepwise increment on the steepness of the aging versus nMVC loss relationship, with high adiposity. Thus, aging‐related changes from the second to the seventh decade were −1.3 versus 0.7 Nm/year in high versus low %body fat categories. However, similar to the BMI classification, this difference in the slope was statistically significant (Student's *t* statistic 4.82; *P* < 0.05; Fig. 5C).

As expected, there was a significant decrease in intrinsic strength with aging (−0.17 Ncm/cm^3^/year, *P* = 0.027). Grouping by BMI, only highlighted a significant change with aging in the overweight (see Table 1). Grouping by adiposity highlighted a significant decrease in the low‐adipose group but no change in the high‐adipose group (see Table 2).

Unexpectedly, there was no change in specific force with aging (*P* > 0.05). Also, grouping by BMI or adiposity highlighted no subgroup effect (see Tables 1 and 2).

## Discussion

This study is the first to systematically quantify the impact of varying levels of BMI and adiposity on skeletal muscle intrinsic (i.e., whole muscle level) and specific (i.e., fascicle level) force. When body composition status was classified by BMI, the obese cohort exhibited greater muscle volume and nMVC. Interestingly, however, the obese (compared to normal weight and underweight individuals) in fact had lower nMVC normalized to muscle volume, as well as lower muscle specific force. This significant trend was repeated when obesity was classified using adiposity, as the high‐adipose female cohort (≥40% body fat) demonstrated both higher muscle volume and nMVC, yet lower nMVC normalized to muscle volume and specific force. These findings supported our first hypothesis.

The rate of aging in terms of both muscle volume and nMVC was found to be worst in the obese cohort whether classified by BMI or adiposity, again supporting one of our hypotheses. However, contrary to our final hypothesis, a decrease in intrinsic strength was only apparent in the pooled population (regardless of obesity status). Where the data were grouped by BMI, only the overweight showed an aging‐associated decrease in intrinsic strength. No aging changes were significant with the population grouped by adiposity. Specific force was not affected by aging in the pooled population nor where the population was grouped either by BMI or adiposity.

### The effect of BMI and adiposity on muscle contractile characteristics

The classification of obesity in the majority of previous studies investigating the effect of high levels of adiposity on muscle structure and function has been through BMI (Hulens et al. 2001, 2002; Rolland et al. 2004; Maffiuletti et al. 2007, 2008; Delmonico et al. 2009). However, BMI does not distinguish between fat, lean, and bone mass in the calculation of obesity and instead utilizes an individual's body mass relative to their height. We argued that this had the potential to conceal the true effect that differing levels of adiposity may have on skeletal muscle properties (Rothman 2008). Therefore, the results of the current study were categorized by both BMI and body fat percentage to determine whether one or the other obesity classification strategy would be the most powerful in distinguishing between the populations (Baumgartner et al. 2004; Rolland et al. 2009).

The two obesity classifications (i.e., by BMI or adiposity) provided comparable conclusions with regard to the reported magnitude of group differences in muscle contractile characteristics between obese and nonobese classes. This single conclusion supports the hypothesis that obesity does indeed loads the antigravity musculature in a manner similar to simulated hypergravity (Bosco et al. 1986; Klentrou et al. 2007) and/or resistance training. Indeed our data shows an increase in both nMVC and muscle volume in the adult, young obese individuals. For instance, if primarily focusing on obesity effect on nMVC (net total muscle strength), there was a significant greater maximum torque whether participants were classified obese by BMI (+29%) or adiposity (+27%). Our results concur with previous research findings of +12% (Hulens et al. 2001) and +16.3% (Maffiuletti et al. 2007) significantly greater absolute knee extensor torque in obese compared with normal weight individuals. However, with the aforementioned two studies, the recorded maximum torque was not corrected for agonist muscle activation or antagonist coactivation, leading to an underestimation of the true torque of the obese individuals. Indeed, young obese adults have been demonstrated to have significantly lower (92% vs. 85%) agonist muscle activation capacity compared to age‐matched normal weight counterparts (Tomlinson et al. 2014).

Similarly, obesity was associated with increased GM muscle volume when compared to normal weight (+71%) and ordinary adipose (+60%) classified females. Arguably, this further supports the idea that obesity in the young adult, potentially loads skeletal muscle similar to progressive resistance training (Erskine et al. 2010). This theorem has been supported by Lafortuna et al. (2013) who demonstrated a positive association between adiposity and lower limb skeletal muscle volume using computed tomography in adult females. However, no other study, prior to our present work, had accurately quantified the musculature involved in the specific joint movement of interest, through accounting for the volume and architecture of said musculature.

While the above two parameters (i.e., nMVC and muscle volume) suggest a positive impact of obesity, when normalizing maximum torque to the individual's muscle volume (intrinsic strength), a maladaptation of the skeletal musculature to obesity becomes evident. This is illustrated both when individuals were classified obese through BMI (−26% lower intrinsic strength) and adiposity (−11% lower intrinsic strength). Our results suggest that at the whole muscle level, young adult obese individuals irrespective of obesity classification are at a disadvantage relative to normal weight individuals. Interestingly, when comparing these results to previous work, there appears to be conflicting reports within the literature. On one hand, some researchers (Hulens et al. 2001) reported MVC knee joint torque normalized to total fat‐free mass to be 6–7% significantly lower in obese females. Those authors hypothesized lower agonist muscle activation as being the physiological basis of this obesity effect. However, other authors (Maffiuletti et al. 2007) reported the differences between obese and non‐obese muscles to disappear when torque was normalized to total fat‐free mass. Arguably, muscle volume is a more accurate measure of muscle size than total fat‐free mass (Akagi et al. 2009), hence direct comparison of the results from the literature is meaningless, potentially explaining the conflicting reports.

While normalizing nMVC to muscle volume gives an accurate depiction of the whole muscle level features (i.e., the intrinsic strength), skeletal muscle specific force describes characteristics at fascicle level. The calculation of skeletal muscle specific force accounts for the physiological and biomechanical determinants of a muscles force generating capacity (Maganaris et al. 2001). The fascicle level results from our current study are in‐line with the above described whole muscle level findings. The obese young females classified by both BMI and adiposity demonstrated significantly lower (−35% and −25%, respectively for each classification method) skeletal muscle specific force compared to normal weight individuals. Since this is the first study to control for the neural, morphological, and biomechanical factors in force generation, there are no data at a fascicle level to compare our data against. Nonetheless, the underlying mechanism may potentially be linked to higher levels of inflammation often measured in obese individuals (Hotamisligil et al. 1995) and an increase in fatty infiltration within the muscle (Hilton et al. 2008), both likely to lower the intrinsic and specific strength potential of the obese skeletal musculature.

### Impact of BMI and/or adiposity on the magnitude of aging‐related sarcopenia and asthenia

When rate of aging was determined by populations for changes in muscle volume, nMVC, intrinsic strength, and specific force, the fastest changes were seen with muscle volume and nMVC (as seen in Fig. 5). However, it was apparent that the greatest effect of combined adiposity and aging was seen in the rate of loss of muscle volume (Fig. 5A and C). One of the mechanisms that can underpin the faster loss of muscle mass may be the cumulative effect of higher inflammation observed in both obese and elderly individuals (Cesari et al. 2004; Park et al. 2005; Schrager et al. 2007; Degens 2010), coupled with a lower anabolic profile both in old age (Bucci et al. 2013) and obesity (Frost et al. 2003; Galli et al. 2012). This enhanced susceptibility to sarcopenia in the obese, emphasizes the deleterious impact of this condition, hence highlighting the importance of maintaining a healthy body composition.

The effect of aging on nMVC was significant in old subject groups. However, there were no significant differences between the slopes of the regressions between obesity categories (see Fig. 5B and D). The importance of this finding stems from a further exacerbation of an existing relatively low strength to body mass ratio in obese individuals (Blimkie et al. 1990; Zoico et al. 2004; Lafortuna et al. 2005; Maffiuletti et al. 2007, 2008), thereby rendering daily functional capacity even more compromised, with the ability to carry out tasks such as rising from a chair or squatting deep to reach items on the floor for instance, quicker to lose. Arguably, a more detailed description of decade‐by‐decade changes on sensitivity to adiposity would be warranted in future studies, as would the additional consideration of ethnicity.

Interestingly, in the current study population, the effect of aging on either intrinsic strength or specific force (when categorized by BMI or adiposity) was as expected, as no significant changes were observed. Notably, however, aging was associated with a significant decrement in intrinsic strength in the pooled population. Our data partially support previous reports that have shown specific force to decrease with age (Morse et al. 2005). Interestingly, resistance training has been shown to increase specific force in the elderly (Morse et al. 2007). Therefore, assuming the additional fat mass seen in obesity chronically loads the skeletal muscle to a degree similar to resistance training, together with the extended proportion of their lifespan as obese individuals (Abdullah et al. 2011), may explain the lack of deleterious aging‐related changes in intrinsic strength and muscle specific force, even in this population. Potentially such an effect would be mediated via a shift in muscle fiber‐type composition toward type II fibers. Indeed such fiber‐type composition bias has previously been reported in obese individuals (Kriketos et al. 1997).

The lack of aging‐related decrease in three out of four BMI or adiposity subgroups in the current population, may also be indicative of a healthy older population, as demonstrated through their health questionnaire data.

## Conclusion

Our study demonstrates for the first time that at both whole muscle and fascicular levels, high BMI or adiposity categories of obesity are associated significantly with lower skeletal muscle contractile capacity in young adults. Interestingly, the aging effect on obese individuals classified by both BMI and adiposity was foremost observed through the loss of muscle tissue content as well as total muscle strength. The dissociation in the aging‐related rate of changes in the BMI and/or adiposity categories meant that in the presence of obesity, aging did not lower skeletal muscle intrinsic strength and/or muscle specific force. While this latter finding warrants further investigations, our results suggest that obesity even where individuals are recreationally active (as in the present study sample), should be targeted using therapies aimed at minimizing sarcopenia and asthenia in later life.

## Acknowledgments

The authors are ever indebted to every one of the participants in this study for their time and adherence to the pretest conditions. Additionally, the authors would like to acknowledge the work of Fay Manning and Adam Bentley for their assistance in data collection. D. Tomlinson is the postgraduate student who carried out the day‐to‐day experiments, data analyses, and produced the first manuscript draft. R. Erskine, K. Winwood, and C. Morse are members of the supervision team for D. Tomlinson and were instrumental in the study design, protocol refinements, and data interpretation. G. Onambele is the director of studies, who trained D. Tomlinson, finalized the study design and protocols, and oversaw data analyses as well as all manuscript drafts.

## Conflict of Interest

None declare.
