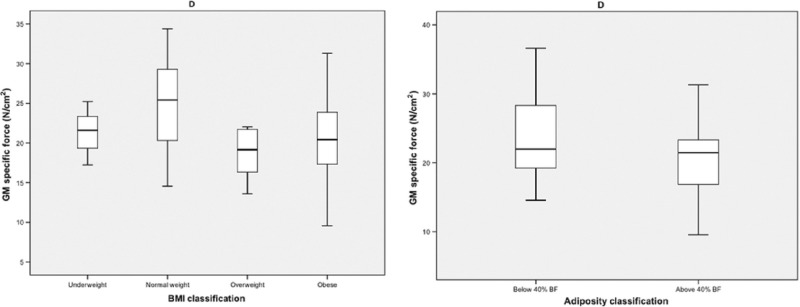# Erratum

**DOI:** 10.14814/phy2.12132

**Published:** 2014-08-19

## Introduction

**Obesity decreases both whole muscle and fascicle strength in young females but only exacerbates the ageing‐related whole muscle level asthenia**

*Physiol Rep*, 2 (6), 2014, e12030, doi: 10.14814/phy2.12030

In the calculation of gastrocnemius medialis (GM) fascicle force, the tendon force was divided by the pennation angle rather than the cosine of the pennation angle, thus leading to an overestimation of GM fascicle force and GM specific force by approximately 38% on average, with heteroscedastic distribution in the individual overestimates.

Thus, we need to refine our concluding remarks to the effect that:

- With BMI classification, obesity did not impact on muscle specific force (*P *= 0.220), (previously specific force was reported as significantly lower in the obese group (*P* < 0.001) compared to that in normal weight counterparts). Please see amended Figures 3D and 4D below.
- With adiposity classification, the results remain as previously reported except that the specific force of the high adiposity group was not significantly lower (*P* = 0.129) than that seen in the low adiposity group (previously this was reported as significantly lower (*P* < 0.01)).
- There was no impact of obesity (either by BMI or adiposity) on the slope of age related changes in terms of specific force.

The lack of impact of obesity on rate of specific force change with increased ageing is complex and warrants further investigations.